# Supplementary material for: Single-atomic platinum on fullerene C60 surfaces for accelerated alkaline hydrogen evolution
Source: Nat Commun. 2023 Apr 28;14:2460. doi: 10.1038/s41467-023-38126-z (PMC10147718; doi:10.1038/s41467-023-38126-z)
Supplement: Supplementary file 1 — Supplementary information [file 41467_2023_38126_MOESM1_ESM.pdf]

Supplementary information

Single-atomic platinum on fullerene C_{60} surfaces for accelerated alkaline hydrogen evolution

Ruiling Zhang,[1] Yaozhou Li,[1] Xuan Zhou,[2] Ao Yu,[1] Qi Huang,[1] Tingting Xu,[1] Longtao Zhu,[1] Ping Peng,[1]* Shuyan Song,[2]* Luis Echegoyen,[3]* Fang-Fang Li[1]*

[1]State Key Laboratory of Materials Processing and Die & Mould Technology, School of Materials Science and Engineering, Huazhong University of Science and Technology, 1037 Luoyu Road, Wuhan 430074, P. R. China, [2]State Key Laboratory of Rare Earth Resource Utilization, Changchun Institute of Applied Chemistry, Chinese Academy of Sciences, 5625 Renmin Street, Changchun 130022, P. R. China, [3]Department of Chemistry and Biochemistry, University of Texas at El Paso, 500 West University Avenue, El Paso, Texas 79968 (USA).

These authors contributed equally: Ruiling Zhang, Yaozhou Li

Supplementary Table 1. ICP-OES analysis for Pt/C_{60}-1, Pt/C_{60}-2 and Pt/C_{60}-4.

Sample	Pt wt%	Ratio of Pt : C_{60}
Pt/C_{60}-1	26.56 wt%	1.335
Pt/C_{60}-2	21.54 wt%	1.013
Pt/C_{60}-4	20.48 wt%	0.951

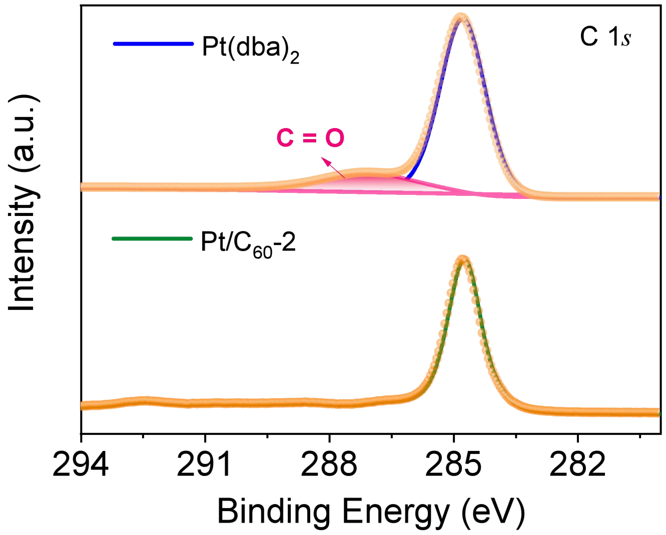

Supplementary Fig. 1. XPS analysis showing the absence of dba ligand in Pt/C_{60}-2. High-resolution XPS C 1*s* spectra of Pt/C_{60}-2 and Pt(dba)$_2$.

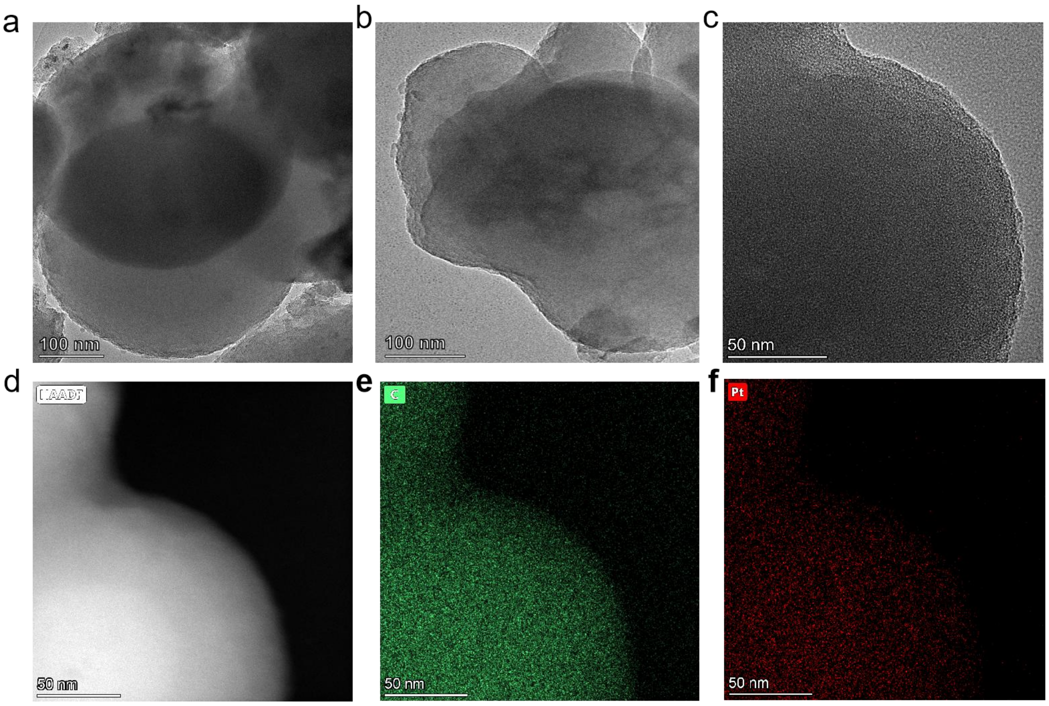

Supplementary Fig. 2. Structure characterizations of Pt/C_{60}-2. a-c TEM images of Pt/C_{60}-2 taken at different domains. **d-f** HAADF-STEM image and the corresponding EDX mapping of Pt/C_{60}-2, showing the presence of Pt and C elements.

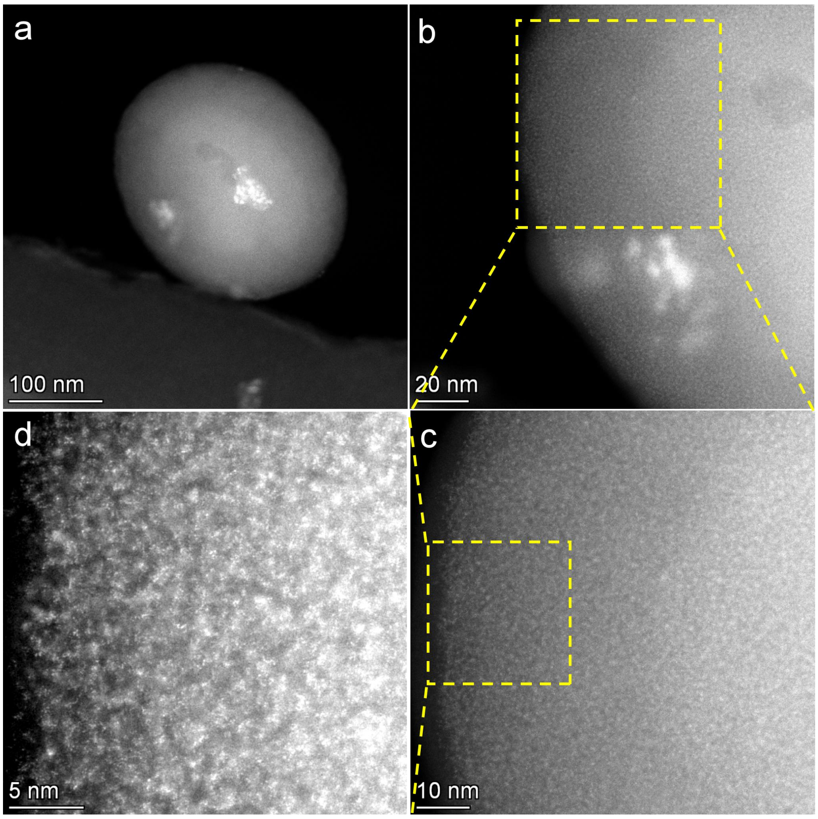

Supplementary Fig. 3. a-d HAADF-STEM analysis of Pt/C_{60}-2. Stepwise observations of the HAADF-STEM images revealed isolated Pt single atoms.

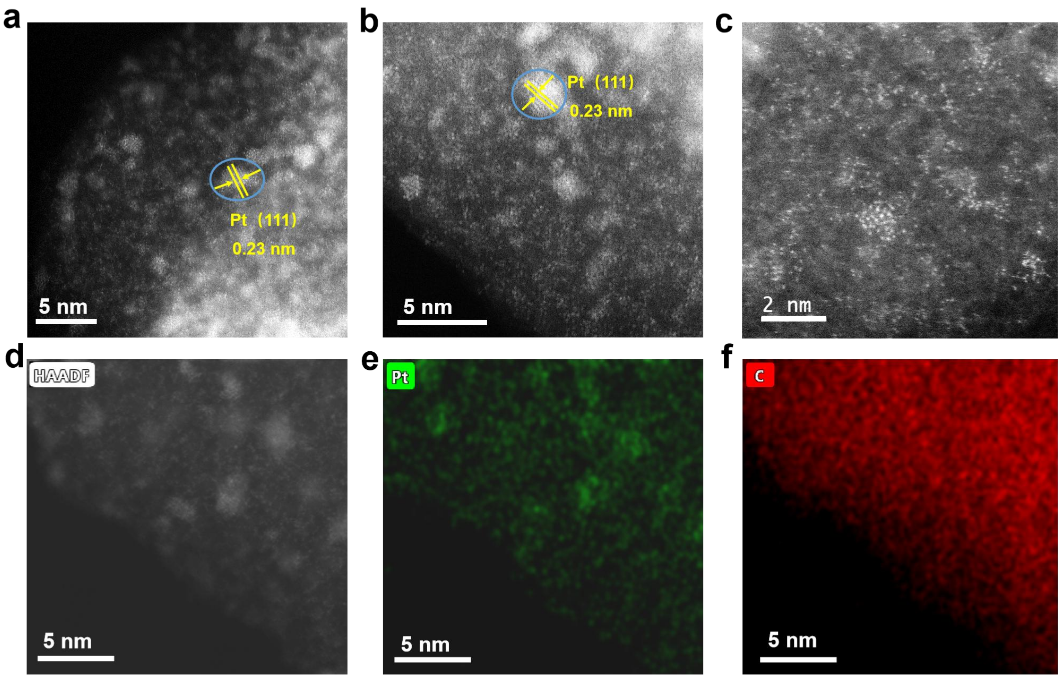

Supplementary Fig. 4. Characterizations of Pt single atoms and Pt clusters. a-c HAADF-STEM images of Pt/C_{60}-2, showing the presence of Pt single atoms and Pt clusters. **d-f** HAADF-STEM image and the corresponding EDX mapping of Pt/C_{60}-2.

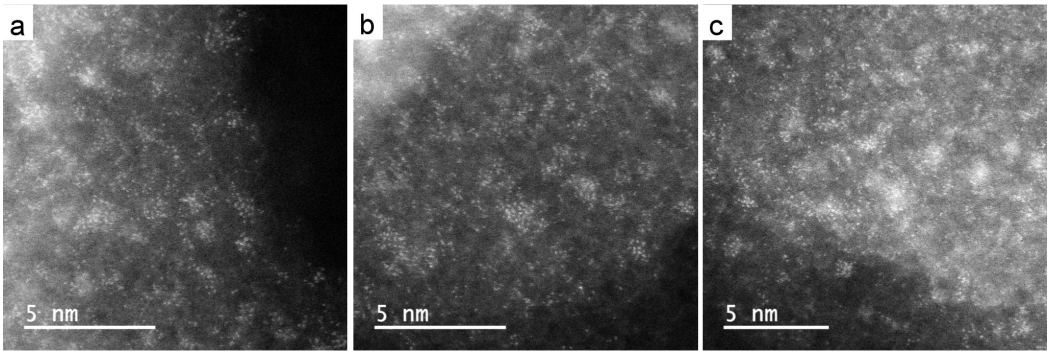

Supplementary Fig. 5. Characterization of atomically dispersed platinum in Pt/C_{60}-2. a-c HAADF-STEM images taken in different regions indicated mainly the presence of atomically dispersed Pt.

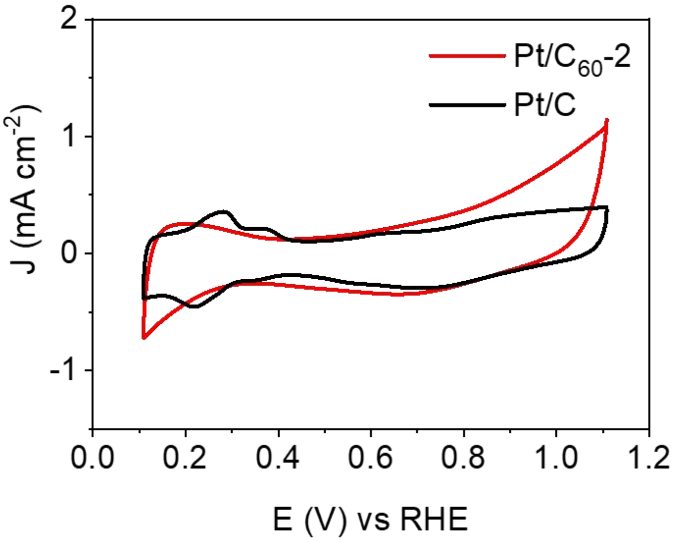

Supplementary Fig. 6. H adsorption-desorption behavior of Pt/C_{60}-2 and Pt/C. The cyclic voltammetry of Pt/C_{60}-2 and Pt/C in N_2-saturated 0.1 M KOH electrolyte at a scan rate of 10 mV s^{-1}.

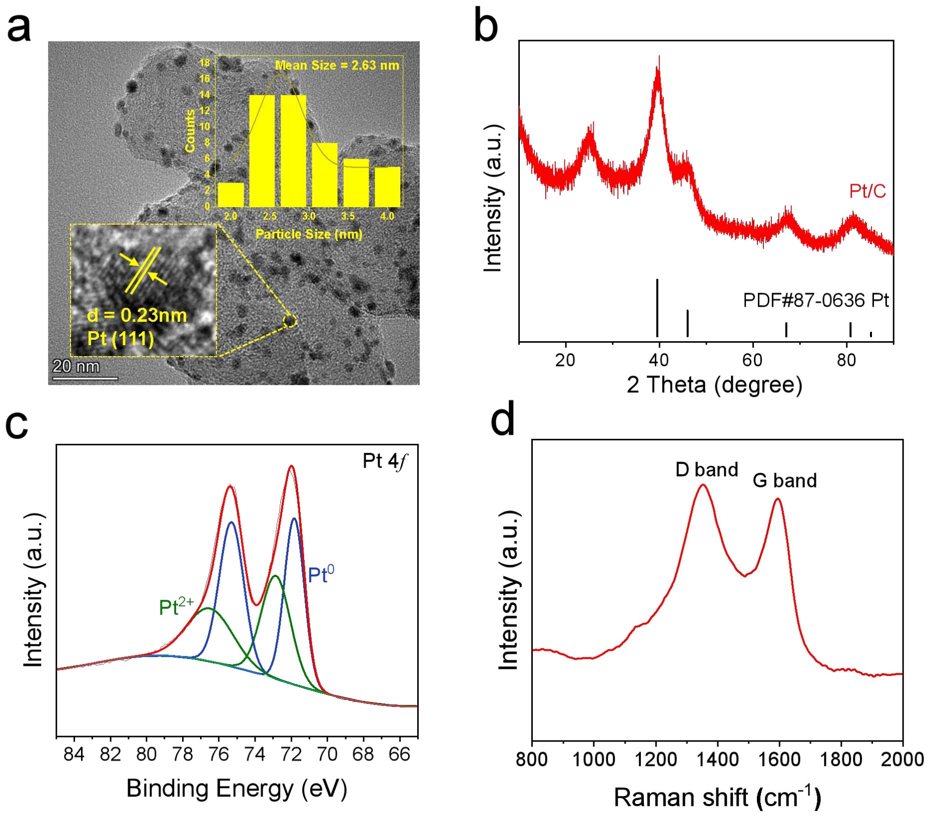

Supplementary Fig. 7. Structure characterizations of the commercial 20 wt% Pt/C. a TEM image, **b** PXRD, **c** XPS, and **d** Raman spectra of the commercial 20 wt% Pt/C used in this study.

Supplementary Table 2. EXAFS fitting parameters for the Pt L_3-edges of the Pt/C_{60}-2 and Pt foil. Here, N represents the coordination number, R represents the bond distance, σ^2 represents the Debye-Waller factor value.

Sample	Path	N	R(Å)	σ^2 (10^{-3} Å^2)
Pt foil	Pt-Pt	12	2.76	0.45
	Pt-Pt	6	3.19	0.45
Pt/C_{60}-2 (SA)	Pt-C	2.52	2.09	0.68

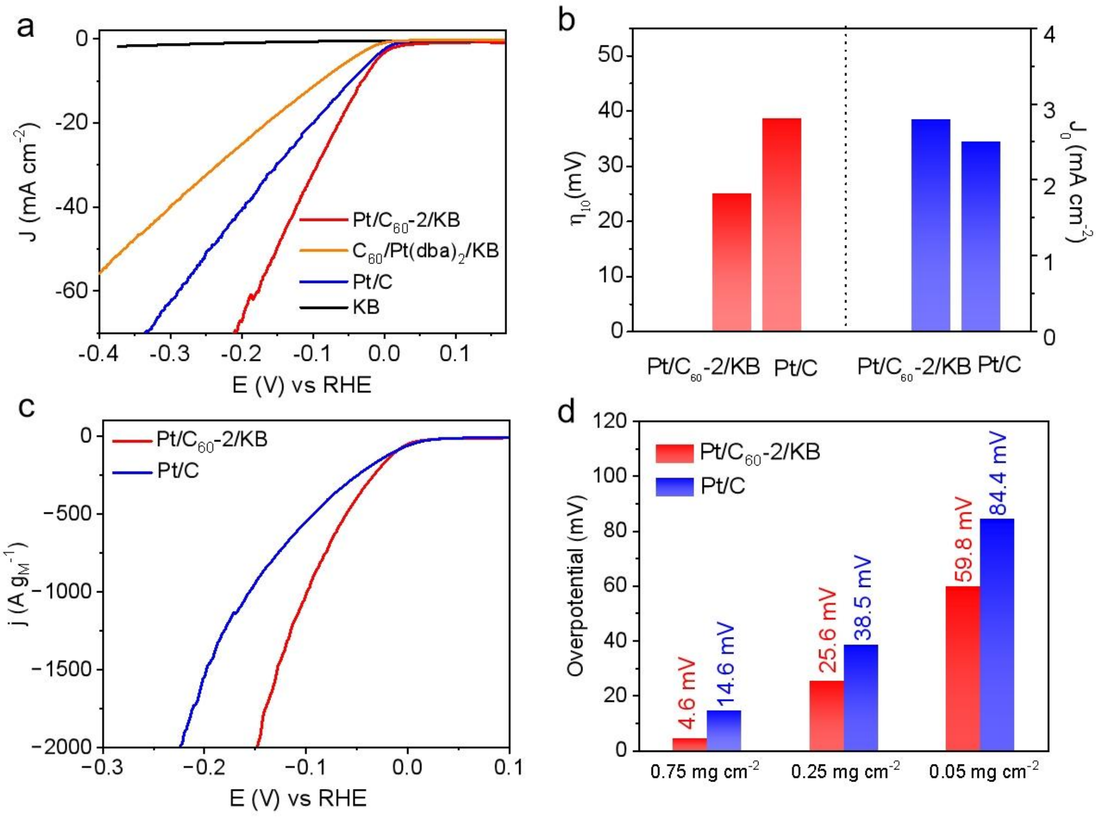

Supplementary Fig. 8. Summary of LSV tests. a HER polarization curves for Pt/C_{60}-2/KB, C_{60}/Pt(dba)$_2$/KB, 20 wt% Pt/C and KB in 1 M KOH without iR correction. **b** η_{10} and j_0 for Pt/C_{60}-2/KB and 20 wt% Pt/C. **c** Mass activity for Pt/C_{60}-2/KB and 20 wt% Pt/C in 1 M KOH. **d** Overpotentials for Pt/C_{60}-2/KB and 20 wt% Pt/C under multiple loadings in 1 M KOH.

Supplementary Table 3. Comparison of the catalytic performance of Pt/C_{60}-2 with other reported Pt-based catalysts in 1 M KOH.

Catalysts	Pt Loading	Overpotential at 10 mA cm^{-2} (mV)	Tafel slope (mV dec^{-1})	Ref.
Pt/C_{60}-2	0.053 mg/cm^2	25	55	This work
Pt/C	0.050 mg/cm^2	39	99	This work
PtRh DNAs	0.126 mg/cm^2	28	47	1
Pt/Ni ASs/C	0.002 mg/cm^2	28	47	2
Pt-SAs/$MoSe_2$	0.028 mg/cm^2	29	41	3
Pt-PdO	0.024 mg/cm^2	29	35.6	4
NF-Na-Fe-Pt	/	31	35.98	5
Pt/MoS_2-NTA/Ti_3C_2	0.348 mg/cm^2	32	35	6
Pt@DG (Pt-C_3)	0.006 mg/cm^2	37	53	7
Pt-WO_{3-x}@rGO	0.0032 mg/cm^2	37	45	8
$Pt_{61}La_{39}$@KB	/	38	/	9
Pt/$PtTe_x$	0.082 mg/cm^2	44	23	10
Ni_3Fe LDH-Pt SAs	0.0194 mg/cm^2	45	54.3	11
PtPd@NLS	0.0014 mg/cm^2	46	124	12
SA In-Pt NWs/C	0.127 mg/cm^2	46	/	13
Pt_5/HMCS	0.0254 mg/cm^2	46.2	48.1	14
Pt-CoP	0.011 mg/cm^2	48	33	15
d-$PtSe_2$	/	59	88	16
Pt-SAs/MoS_2	0.07 mg/cm^2	59	31	17
Pt/$LiCoO_2$	0.0612 mg/cm^2	61	39.5	18
Pt/MOF-O	0.0204 mg/cm^2	66.1	101.6	19
Pt/Zn-P NSs	/	74	55	20
Pt-MoS_2/MWCNTs	0.016 mg/cm^2	75	41	21
NiFe-LDH-Pt-ht/CC	0.003 mg/cm^2	101	127	22
Pt_1-Mo_2C-C	0.0014 mg/cm^2	155	64	23

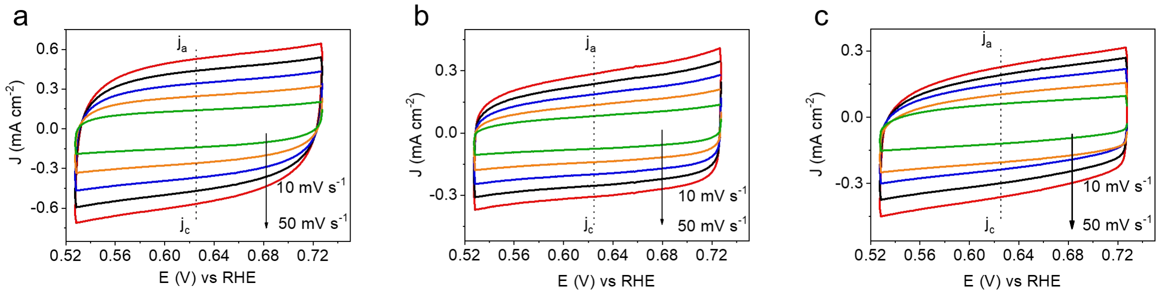

Supplementary Fig. 9. C_{dl} measurements. CV curves at different scan rates for **a** Pt/C_{60}-2/KB, **b** Pt/C and **c** C_{60}/Pt(dba)$_2$/KB in 1 M KOH.

Supplementary Table 4. Comparison of the TOF values of Pt/C_{60}-2 with Pt/C and other reported catalysts in 1.0 M KOH.

Electrocatalysts	Overpotential (mV)	TOF (H_2 s^{-1})	Ref.
	50	2.17	
Pt/C_{60}-2	100	5.55	
	150	11.2	This work
	50	1.37	
20 wt% Pt/C	100	2.95	
	150	5.04	
Pt@DG	100	6.74	7
Pt-SAs/MoS_2	50	1.02	24
Pt-SAs/WS_2	150	6.41	24
Pt/$LiCoO_2$	200	2.25	18
Pt/$PtTe_x$	50	1.43	10
Pt-PdO	100	1.42	4
Ru SAs-Ni_2P	190	3	25
E-Co SAs	100	0.48	26
α-Mo_2C	250	2.5	27
Ru@C_2N	50	1.66	28
Pt_1SAC-VNGNMAs	100	4.1	29
RhPd-H NPs	60	0.33	30
Ir@CON	50	0.66	31
RuCo	50	1.5	32
Ru_{NP}@RuN_x-OFC/NC	100	0.49	33

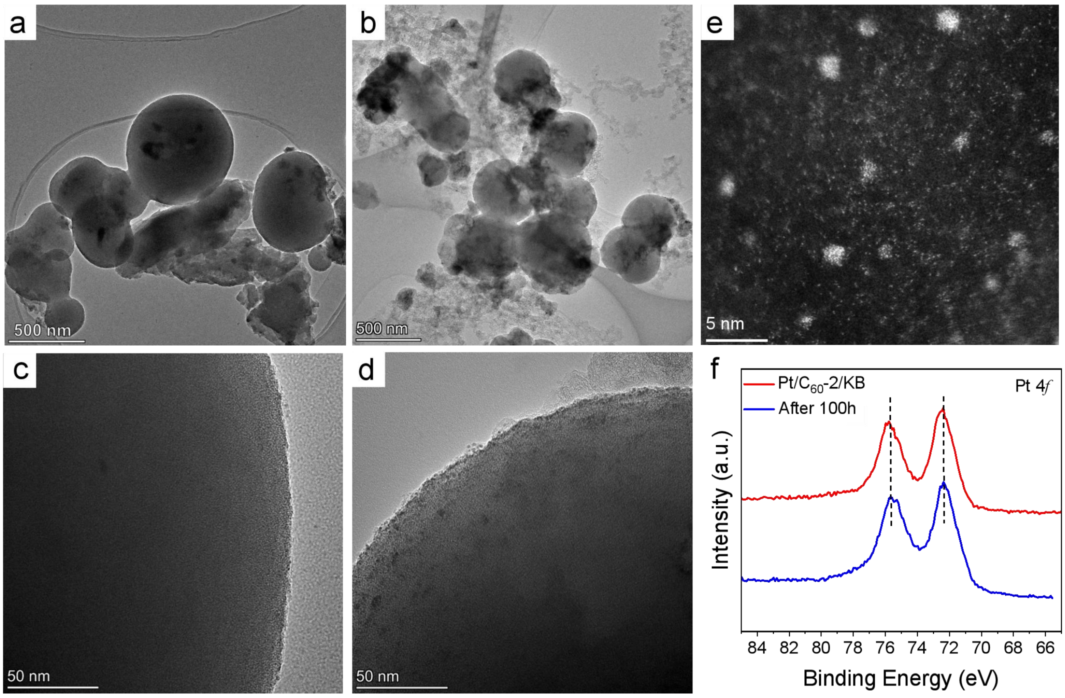

Supplementary Fig. 10. **Structure characterizations of Pt/C_{60}-2 after stability test.** TEM images of Pt/C_{60}-2 before **a,c** and after **b,d** 100 h long-term stability test. **e** HAADF-STEM image of the catalyst after 100 h durability test. **f** XPS spectra of the catalyst before and after the durability test.

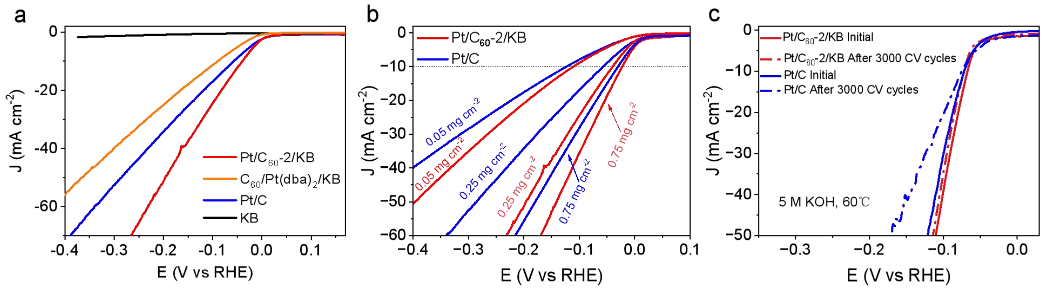

Supplementary Fig. 11. Non-iR corrected LSV curves **a** for Pt/C_{60}-2/KB, C_{60}/Pt$(dba)_2$/KB, Pt/C and KB in 1 M KOH, **b** for Pt/C_{60}-2/KB and Pt/C under multiple loadings in 1 M KOH, **c** for Pt/C_{60}-2/KB and Pt/C before and after 3000 catalytic cycles in 5 M KOH at 60°C.

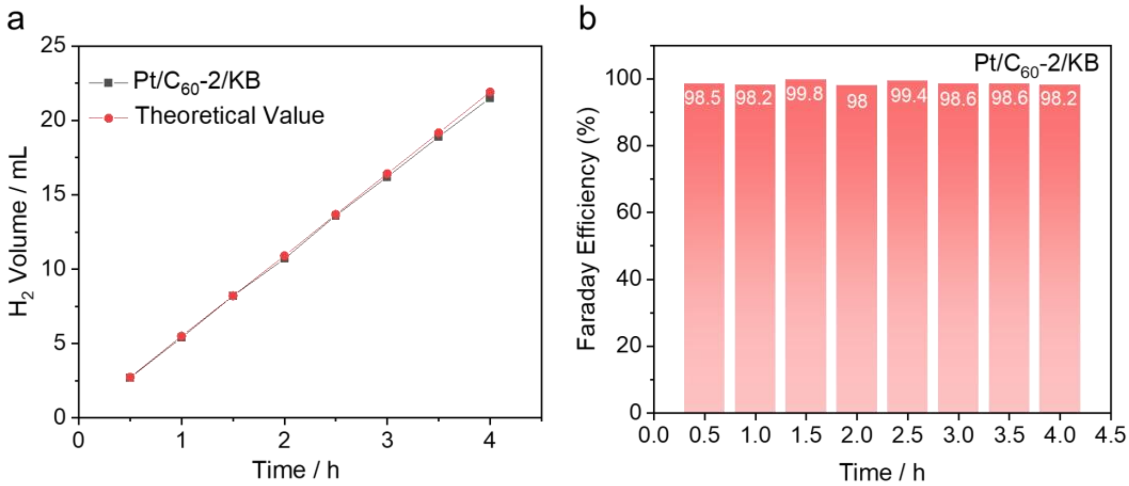

Supplementary Fig. 12. Faraday efficiency measurement. a Theoretical and experimentally collected H_2 volumes. **b** Faraday efficiency for Pt/C_{60}-2.

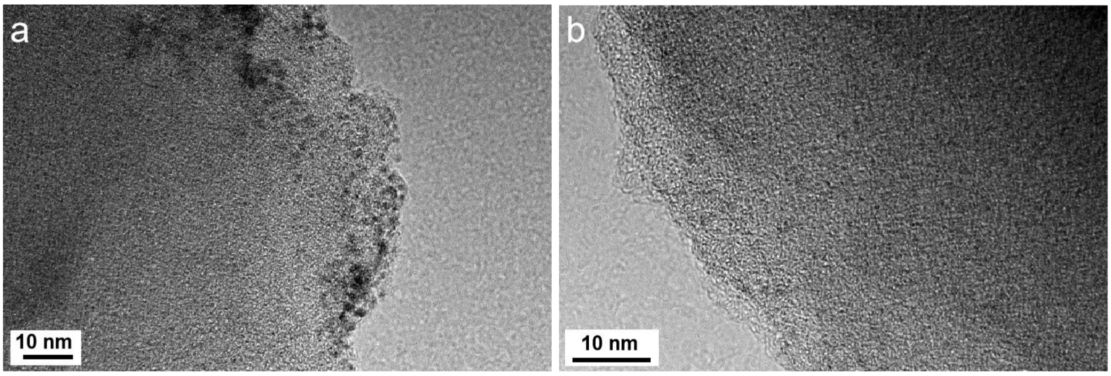

Supplementary Fig. 13. Structure characterizations of Pt/C_{60}-1 and Pt/C_{60}-4. TEM images of **a** Pt/C_{60}-1 and **b** Pt/C_{60}-4 over a wider area, showing more abundant Pt clusters in Pt/C_{60}-1.

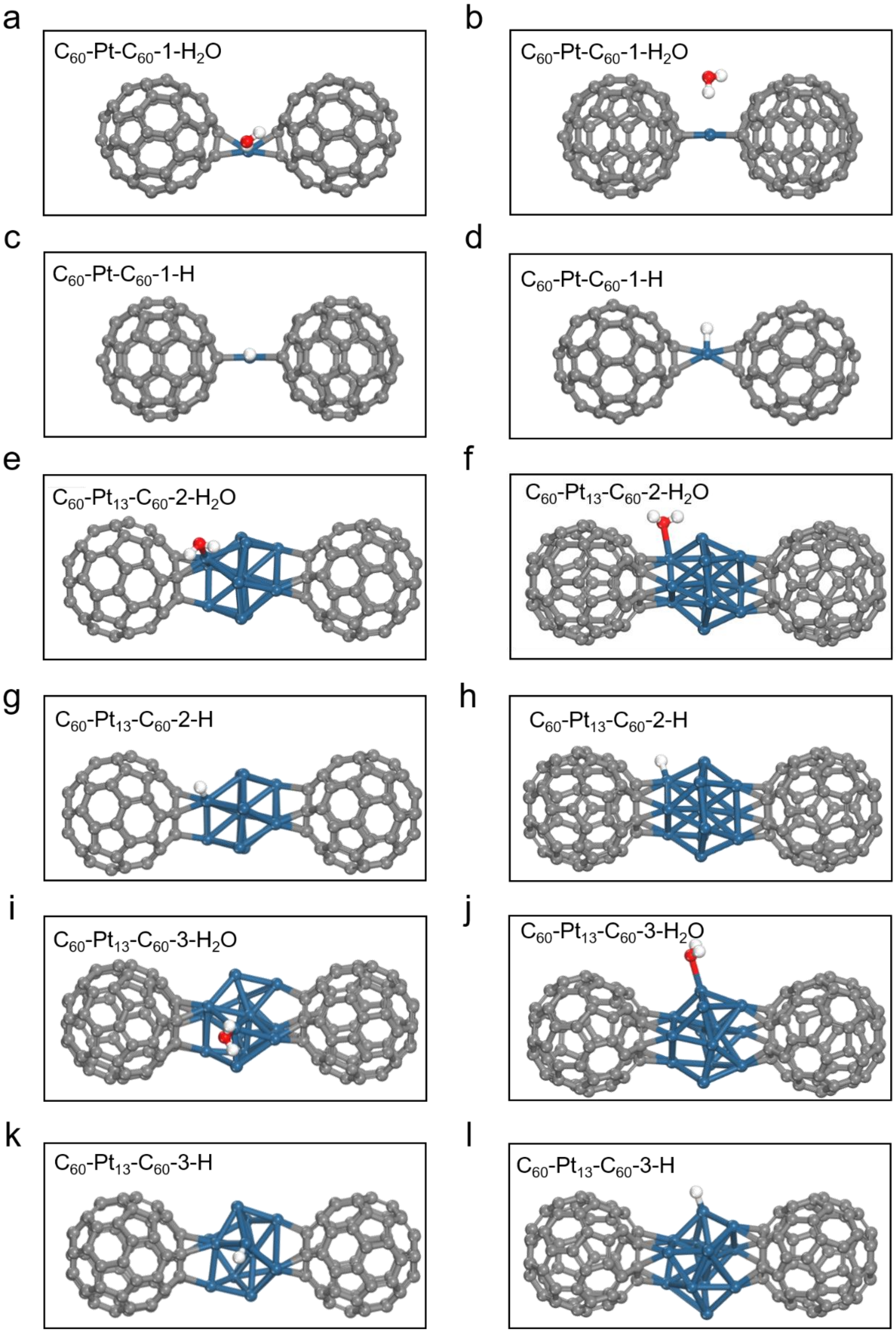

Supplementary Fig. 14. Structure Models for H_2O and H adsorption. The top and side views of **a-b** C_{60}-Pt-C_{60}-1-H_2O, **c-d** C_{60}-Pt-C_{60}-1-H, **e-f** C_{60}-Pt_{13}-C_{60}-2-H_2O, **g-h** C_{60}-Pt_{13}-C_{60}-2-H, **i-j** C_{60}-Pt_{13}-C_{60}-3-H_2O, **k-l** C_{60}-Pt_{13}-C_{60}-3-H.

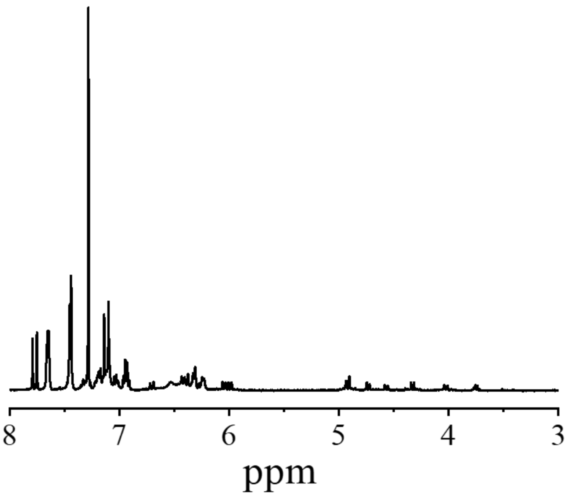

Supplementary Fig. 15. Structure characterization of Pt(dba)$_2$. ^{1}H NMR spectrum of Pt(dba)$_2$ recorded in $CDCl_3$ at 600 MHz.

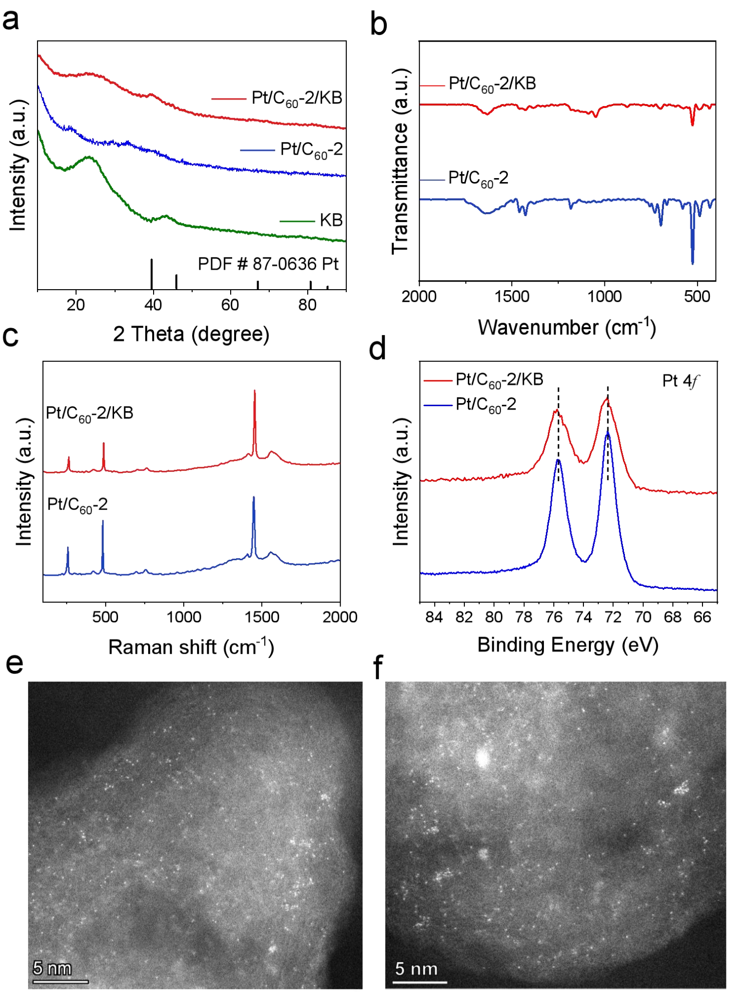

Supplementary Fig. 16. Structure characterizations of the ink sample. a PXRD, **b** FT-IR, **c** Raman, and **d** XPS spectra of the ink sample (Pt/C_{60}-2/KB) and pristine Pt/C_{60}-2. **e-f** HAADF-STEM images of ink.

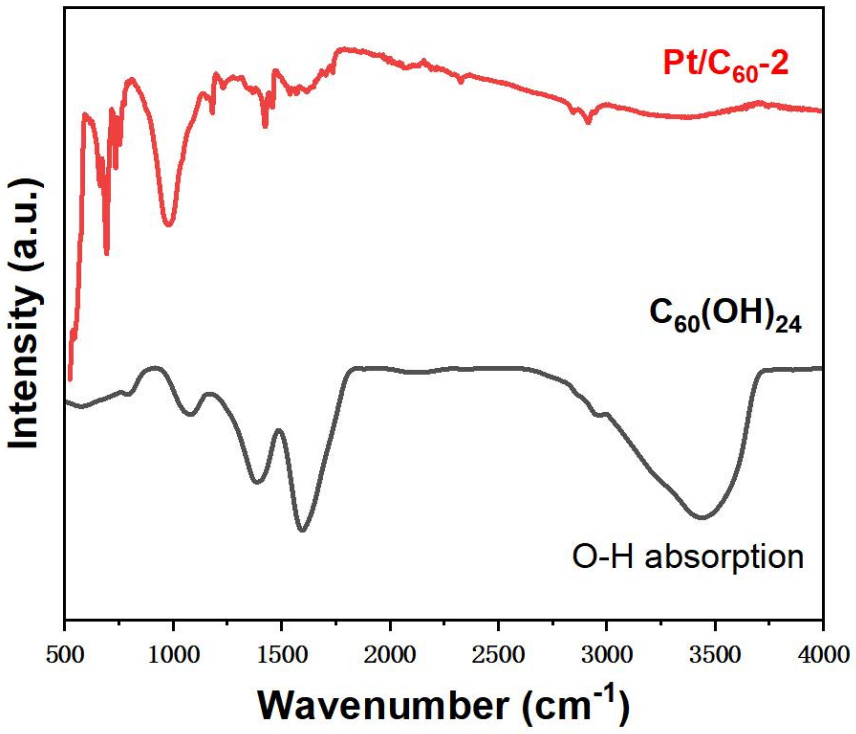

Supplementary Fig. 17. Tolerance of Pt/C_{60}-2 to 1 M KOH. FT-IR spectra of KOH-treated Pt/C_{60}-2 and $C_{60}(OH)_{24}$.

Supplementary References

1. Han, Z., Zhang, R.-L., Duan, J.-J., Wang, A.-J.,Zhang, Q.-L. Platinum-rhodium alloyed dendritic nanoassemblies: An all-pH efficient and stable electrocatalyst for hydrogen evolution reaction. *Inter. J. Hydro. Energy* **45**, 6110-6119 (2020).
2. Ding, J., Ji, Y., Li, Y.,Hong, G. Monoatomic platinum-embedded hexagonal close-packed nickel anisotropic superstructures as highly efficient hydrogen evolution catalyst. *Nano Lett.* **21**, 9381-9387 (2021).
3. Shi, Y., Ma, Z., Xiao, Y.,Yin, Y. Electronic metal-support interaction modulates single-atom platinum catalysis for hydrogen evolution reaction. *Nat. Commun.* **12**, 3021 (2021).
4. Samanta, R., Mishra, R.,Barman, S. Interface-engineered porous Pt–PdO nanostructures for highly efficient hydrogen evolution and oxidation reactions in base and acid. *ACS Sustain. Chem. & Eng.* **10**, 3704-3715 (2022).
5. Zhao, Y., Gao, Y., Chen, Z., Li, Z., Ma, T.,Wu, Z. Trifle Pt coupled with NiFe hydroxide synthesized via corrosion engineering to boost the cleavage of water molecule for alkaline water-splitting. *Appl. Catal. B: Environ.* **297**, 120395 (2021).
6. Jiao, S., Kong, M., Hu, Z., Zhou, S., Xu, X.,Liu, L. Pt atom on the wall of atomic layer deposition (ALD)-made MoS_2 nanotubes for efficient hydrogen evolution. *Small* **18**, e2105129 (2022).

7. Yang, Q., Liu, H., Yuan, P.,Jia, Y. Single carbon vacancy traps atomic platinum for hydrogen evolution catalysis. *J. Am. Chem. Soc.* **144**, 2171-2178 (2022).
8. Yin, D., Cao, Y., Chai, D.,Fan, L. A WO_x mediated interface boosts the activity and stability of Pt-catalyst for alkaline water splitting. *Chem. Eng. J.* **431**, 133287 (2022).
9. Nie, N., Zhang, D., Wang, Z., Qin, Y., Zhai, X., Yang, B., Lai, J.,Wang, L. Superfast synthesis of densely packed and ultrafine Pt-Lanthanide@KB via solvent-free microwave as efficient hydrogen evolution electrocatalysts. *Small* **17**, e2102879 (2021).
10. Chen, J., Qin, M., Ma, S., Fan, R.,Zheng, X. Rational construction of $Pt/PtTe_x$ interface with optimal intermediate adsorption energy for efficient hydrogen evolution reaction. *Appl. Catal. B: Environ.* **299**, 120640 (2021).
11. Chen, W., Wu, B., Wang, Y.,Zhou, W. Deciphering the alternating synergy between interlayer Pt single-atom and NiFe layered double hydroxide for overall water splitting. *Energy & Environ. Sci.* **14**, 6428-6440 (2021).
12. Wen, F., Zhang, Y., Tan, J., Zhou, Z.,Zhu, M. Pt-Pd co-electrodeposited nitrogenous loofah sponge as efficient pH-universal electrocatalyst for hydrogen evolution reaction. *J. Electroanal. Chem.* **822**, 10-16 (2018).
13. Zhu, Y., Zhu, X., Bu, L.,Shao, Q. Single-atom In-doped subnanometer Pt nanowires for simultaneous hydrogen generation and biomass upgrading. *Adv. Funct. Mater.* **30**, 2004310 (2020).
14. Wan, X., Wu, H., Guan, B., Luan, D.,Lou, X. Confining sub-nanometer Pt clusters in hollow mesoporous carbon spheres for boosting hydrogen evolution activity. *Adv. Mater.* **32**, e1901349 (2020).
15. Jiang, Z., Ren, J., Li, Y., Zhang, X.,Zhang, P. Low-cost high-performance hydrogen evolution electrocatalysts based on Pt-CoP polyhedra with low Pt loading in both alkaline and neutral media. *Dalton Trans.* **48**, 8920-8930 (2019).
16. Chang, Y., Zhai, P., Hou, J.,Zhao, J. Excellent HER and OER catalyzing performance of Se-vacancies in defects-engineered $PtSe_2$: from simulation to experiment. *Adv. Energy Mater.* **12**, 2102359 (2021).
17. Shi, Y., Huang, W.M., Li, J., Zhou, Y., Li, Z.Q., Yin, Y.C.,Xia, X.H. Site-specific electrodeposition enables self-terminating growth of atomically dispersed metal catalysts. *Nat. Commun.* **11**, 4558 (2020).
18. Zheng, X., Cui, P., Qian, Y., Zhao, G.,Zheng, X. Multifunctional active-center-transferable platinum/lithium cobalt oxide heterostructured electrocatalysts towards superior water splitting. *Angew. Chem. Int. Ed.* **59**, 14533–14540 (2020).
19. Wang, M., Xu, Y., Peng, C., Chen, S.,Lin, Y. Site-specified two-dimensional heterojunction of Pt nanoparticles/metal-organic frameworks for enhanced hydrogen evolution. *J. Am. Chem. Soc.* **143**, 16512-16518 (2021).
20. Mai, L., Lam, T., Bui, Q.,Nhac-Vu, H. Efficient hydrogen evolution reaction in alkaline via novel hybrid of Pt deposited zinc phosphide nanosheets. *Mater. Res. Bull.* **133**, 111024 (2021).

21. Fan, A., Zheng, P., Qin, C., Zhang, X.,Dai, X.P. Few-layer MoS_2 and Pt nanoparticles Co-anchored on MWCNTs for efficient hydrogen evolution over a wide pH range. *Electrochim. Acta* **358**, 136927 (2020).
22. Anantharaj, S., Karthick, K., Venkatesh, M.,Simha, T.V.S.V. Enhancing electrocatalytic total water splitting at few layer Pt-NiFe layered double hydroxide interfaces. *Nano Energy* **39**, 30-43 (2017).
23. Niu, S., Yang, J., Qi, H., Su, Y.,Wang, Z. Single-atom Pt promoted Mo_2C for electrochemical hydrogen evolution reaction. *J. Energy Chem.* **57**, 371-377 (2021).
24. Shi, Y., Ma, Z.-R., Xiao, Y.-Y., Yin, Y.-C.,Huang, W.-M. Electronic metal–support interaction modulates single-atom platinum catalysis for hydrogen evolution reaction. *Nat. Commun.* **12**, 3021 (2021).
25. Wu, K., Sun, K., Liu, S., Cheong, W.-C.,Chen, Z. Atomically dispersed Ni–Ru–P interface sites for high-efficiency pH-universal electrocatalysis of hydrogen evolution. *Nano Energy* **80**, 105467 (2021).
26. Liu, X., Zheng, L., Han, C., Zong, H.,Lee, H. Identifying the activity origin of a cobalt single-atom catalyst for hydrogen evolution using supervised learning. *Adv. Funct. Mater.* **31**, 2100 (2021).
27. Ma, L., Ting, L., Molinari, V., Giordano, C.,Yeo, B.S. Efficient hydrogen evolution reaction catalyzed by molybdenum carbide and molybdenum nitride nanocatalysts synthesized via the urea glass route. *J. Mater. Chem. A* **3**, 8361-8368 (2015).
28. Mahmood, J., Li, F., Jung, S.-M., Okyay, M.S.,Ahmad, I. An efficient and pH-universal ruthenium-based catalyst for the hydrogen evolution reaction. *Nat. Nanotech.* **12**, 441-446 (2017).
29. Chi, K., Chen, Z., Xiao, F., Guo, W.,Xi, W. Maximizing the utility of single atom electrocatalysts on a 3D graphene nanomesh. *J. Mater. Chem. A* **7**, 15575-15579 (2019).
30. Fan, J., Cui, X., Yu, S., Gu, L.,Zhang, Q. Interstitial hydrogen atom modulation to boost hydrogen evolution in Pd-based alloy nanoparticles. *ACS Nano* **13**, 12987-12995 (2019).
31. Mahmood, J., Anjum, M., Shin, S.H.,Ahmad, I. Encapsulating Iridium nanoparticles inside a 3D cagemmike organic network as an efficient and durable catalyst for the hydrogen evolution reaction. *Adv. Mater.* **30**, 1805606 (2018).
32. Mao, J., He, C.-T., Pei, J., Chen, W.,He, D. Accelerating water dissociation kinetics by isolating cobalt atoms into ruthenium lattice. *Nat. Commun.* **9**, 4958 (2018).
33. Feng, Y., Feng, W., Wan, J., Chen, J., Wang, H.,Li, S. Spherical vs. planar: Steering the electronic communication between Ru nanoparticle and single atom to boost the electrocatalytic hydrogen evolution activity both in acid and alkaline. *Appl. Catal. B: Environ.* **307**, 121193 (2022).
